# What makes a frontal area of primate brain the frontal eye field?

**DOI:** 10.3389/fnint.2015.00033

**Published:** 2015-05-18

**Authors:** Gérard Percheron, Chantal François, Pierre Pouget

**Affiliations:** Sorbonne Universités, UPMC Univ Paris 06, INSERM, CNRS, UM75, U1127, UMR 7225, ICMParis, France

**Keywords:** FEF, saccades, attention, anatomy, comparative, cortex

## Abstract

The frontal eye field region (FEF) of the oculomotor pathways has been intensely studied. The primary goal of this review is to illustrate the phylogenetic displacement of the FEF locus in primate species. The locus is arrayed along the arcuate sulcus in monkeys and abuts into the primary motor strip region in humans. The strengths and limitations of the various functional, anatomical and histological methodologies used to identify such regions are also discussed.

## Introduction

Since the earliest studies of the brain as an organ, many criteria have been used to define subtypes and hierarchical organization within and between distinct brain regions. Historically, this has meant describing configurations of multiple brain areas believed to be associated with distinct functions and/or sub-functions according to anatomical landmarks. However, functional classifications have also been widely developed to allow existing definitions of anatomical brain regions to be questioned and reexamined (Brodmann, 1909; von Economo and Koskinas, 1925; Kononova, 1949, 1955; Bailey and von Bonin, 1951; Sarkissov et al., 1955; Sanides, 1964).

Classical definitions of brain region are based on many cytoarchitectonic and comparative anatomical studies. One important criterion of classification is the delineation of areas whose neurons have been retrogradely labeled after injecting another distinct region in the brain (Kitai and Bishop, 1981; Mesulam, 1982). This represents *a connective criterion*. This connectivity criterion has been used, for example, to define the axons projecting from the motor cortex to downstream motor centers. Another long-established tool that remains valuable is the delineation of zones where low-threshold currents produce functional modulation, for example evoked movements or perceptions. These represent *functionally evoked criteria*. In primates, such criteria have been used, for example, to define the region of the FEF by evoking eye movement while stimulating regions rostral to the arcuate sulci (Ferrier, 1874; Foerster, 1931). More recently, and particularly in humans, novel functional imaging methods relying on different criteria, such as changes in blood flow, diffusion of water molecules, or other concepts have been used (Price, 2012). The primary goal of this review is to illustrate the phylogenetic displacement of the FEF locus in primate species. We will illustrate our reasoning with a discussion of the location of the FEF in different primate brains according to various anatomical and functional criteria collected in recent decades in different primate brains (see for example Huerta et al., 1987; Tehovnik et al., 2000; Amiez and Petrides, 2009).

Some of this discussion will be based on the hitherto unpublished work of Dr. Gérard Percheron, who died in January 2011. His notes have been revisited and translated as part of the preparation of this review. A neurologist by training, and a former intern in Paris hospitals, Percheron had an early passion for basal ganglia morphology. He was one of the founders of International Basal Ganglia society, first established at a 1983 meeting in Lorne, in the state of Victoria, Australia. His enthusiasm for the study of the thalamus endured, and he described a functionally oriented partitioning of the thalamus in primates. Upon retirement, he continued to write on topics related to the anatomical organization of the cerebral cortex, with a particular interest in phylogenetic development across the primate order.

### The Architectonic Criteria and Position in Relation to Sulci

**Figure 1:**
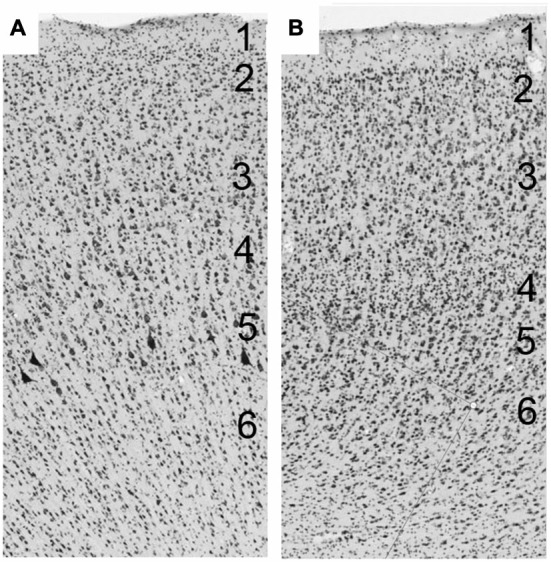
**(A)** The cerebral cortex is a layered structure consisting of up to six horizontal layers perpendicular to the superficial laminae. **(A)** Laminar organization of the primary motor cortex of macaque. **(B)** Laminar organization of the frontal eye field (FEF) of macaque.

At the beginning of the 20th century, cortical architectonic studies began to attempt to characterize histologic entities according to their function. Various cytoarchitectonic (Brodmann, 1909; von Economo and Koskinas, 1925; Kononova, 1949, 1955; Bailey and von Bonin, 1951; Sarkissov et al., 1955; Sanides, 1964) and myeloarchitectonic (Campbell, 1905; Vogt, 1927; Strasburger, 1937; Filimonoff, 1949; Sanides, 1964) criteria enabled the division of the cerebral cortex into “areas”. The cortex is classically characterized as a stratified or laminated neuronal assembly, whose parallel, superimposed layers allow organization perpendicular to the superficial laminae, and make columnar differentiation possible (Figure 1). Most regions of the cerebral cortex consist of six laminae (isocortex), with some variations and exceptions. This six-layer cerebral cortex is referred to as homotypical or eulaminate—the so-called association cortex (Brodmann, 1909; von Economo and Koskinas, 1925; von Economo, 1927; von Bonin and Bailey, 1947; Bailey and von Bonin, 1951). Heterotypic cortex refers to areas possessing fewer than six layers: the so-called granular and agranular types. The granular type (or koniocortex) is found in sensory cortices and is characterized by closely packed non-pyramidal cells in layers II–IV, which make it difficult to distinguish the layers in these areas. Afferent cortical fibers synapse in layers II and IV, with layer IV being the privileged stage of reception of specific thalamic axonal terminations. The agranular type is found in various regions, such as in the anterior part of the cingulate cortex, which lacks a granular layer IV, and the motor cortex, though a distinct interneuron layer IV has been recently and very elegantly described by Garcia-Cabezas in the motor cortices (Garcia-Cabeza and Barbas, 2014). Efferent cortical fibers exit from layers III and V (White, 1989; Snell, 1997). The dysgranular cortex is the cortical region that is transitional between the agranular and the granular cortex (von Bonin and Bailey, 1947). After von Economo (1927), we consider gyral parts to be superficial in gyri, sulcal parts to be located on the banks of the sulci, and fundic parts to be located in the depths of the sulcus.

### The Histotype and Anatomy of the Frontal Eye Field Region

#### Cercopithecidae

Old World monkeys (Cercopithecidae) are a group of simians native to Old World regions including Africa, India and Southeast Asia. Old World monkeys are medium to large in size. Some species are arboreal while others are terrestrial. Cercopithecids are almost exclusively diurnal. Most of the anatomical studies on area 8 have been carried out on Cercopithecidae, which includes the diverse genera of the macaques. It is now more than a century since the oculomotor cortical areas in Cercopithecidae were first discovered and described. The cerebral sulcal pattern is very stable across the whole family (Falk, 1978), with an obvious and deep arcuate sulcus and a straight sulcus principalis or rectus. The oculomotor cortex is mainly gyral but also partially sulcal (Brodmann, 1905). Most investigators, including Brodmann, have recognized at least two cytoarchitectonic areas on this gyrus. Walker (1940) distinguished area 8a close to the superior branch of the arcuate sulcus and area 45 (along the inferior part), which is a part of area 8 (Brodmann, 1905; Vogt and Vogt, 1919). Area 8 is characterized by a thin but evident granular layer (Mauss, 1908) with large pyramidal cells in layers III and V (Brodmann, 1905; von Bonin and Bailey, 1947). Briefly, the oculomotor cortex of Cercopithecidae is mainly gyral, partially sulcal, *and granular*.

**Figure 2:**
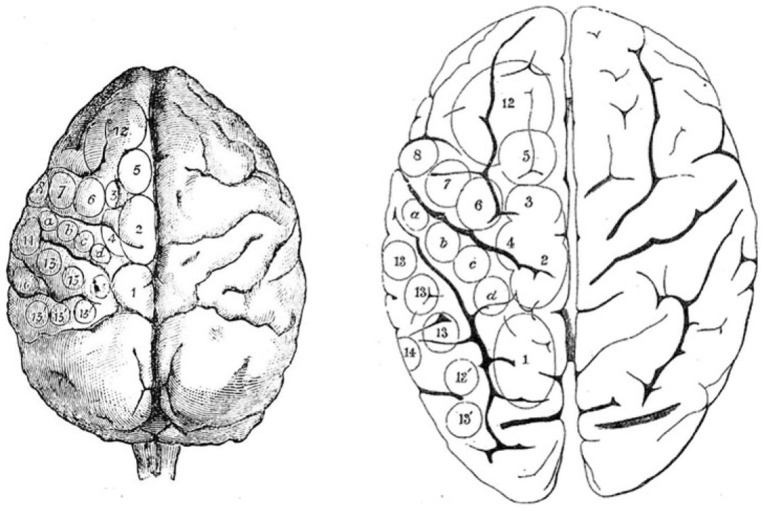
**Ferrier’s projection of areas on monkey brain (left) from which he used to guide his study of stimulation of the human brain (right)**. From Ferrier (1874).

The oculomotor effect of cortical stimulation described by Ferrier (1874, 1875), Beevor and Horsley (1888), Mott and Schaefer (1890), and Vogt and Vogt (1907) was found to be the most effective just anterior to the arcuate sulcus (Figure 2). All stimulation studies since agree that the frontal oculomotor cortex has its core in the inferior arcuate sulcus but some (probably depending on stimulation parameters) delineated a wider area. Smith (1936, 1949) described an oculomotor area extending dorsally (to upper 6 and 9). This was reduced by Crosby (1953) and Brucher (1955, 1964), extended by Robinson and Fuchs, even more restricted by Bruce et al. (1985) (to the posterior portion of the arcuate sulcus, and mainly its anterior bank), before being re-extended again by Moschovakis et al. (2004). What is most commonly named today in macaques as the FEF (Huerta et al., 1986) mainly corresponds to cytoarchitectonic area 8 or FDΓ. FEF or area 8 (if they are the same) has been functionally subdivided into two parts (though this distinction was not retained by Huerta et al., 1986): one dealing with pursuit and the other with saccades (Bruce et al., 1985). The saccadic region is located in a restricted area along the anterior wall of the arcuate sulcus, whereas the pursuit part is located deeper in the sulcus close to the fundus (Fukushima et al., 1999, 2008).

#### Cebidae

**Figure 3:**
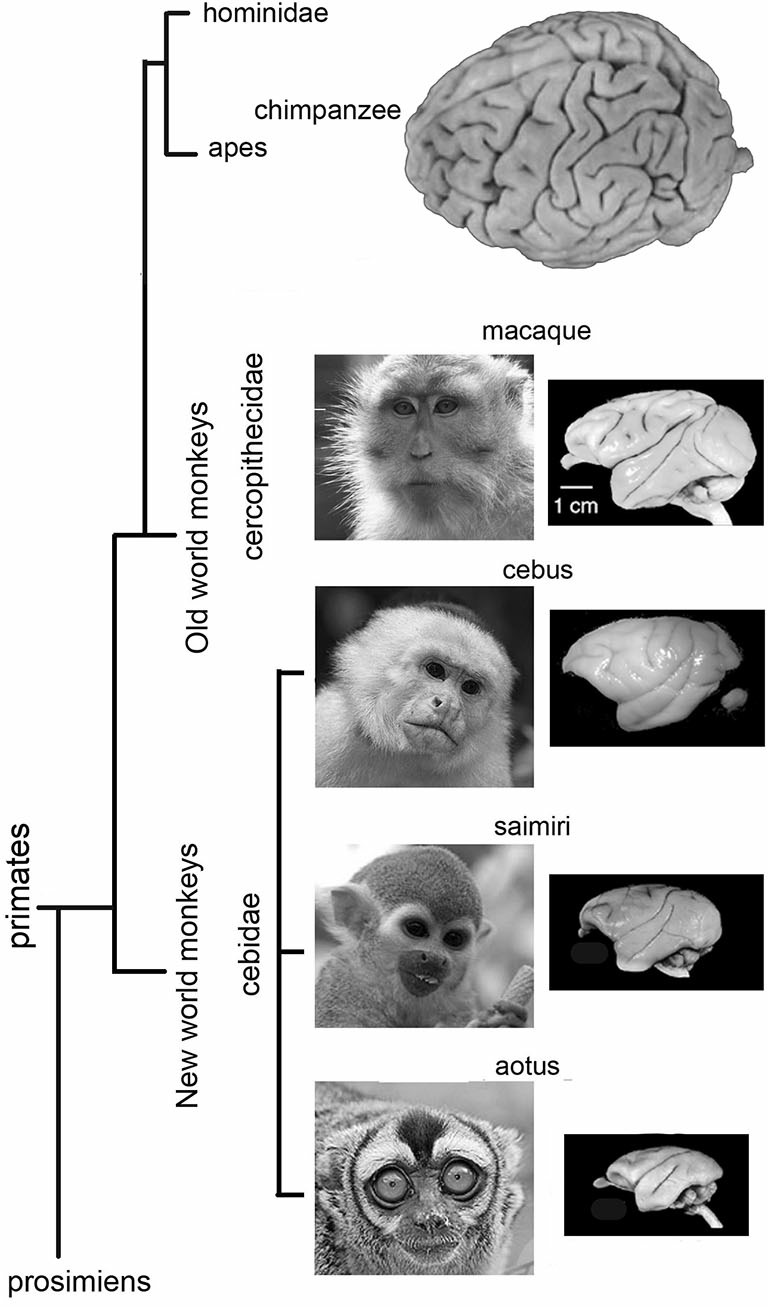
**Lateral views of Owl monkey (*Aotus trivarigatus*), Squirrel monkey (*Saimiri sciureus*), *Cebus* monkey (*apella* or unspecified) and Macaque monkey brains**. Note that the brain of Owl monkey is mostly lissencephalic anterior to the central sulcus. The gyral variations of squirrel monkey ranges from no sulcus at all to a clear gyral pattern. Note that the gyral pattern is close to that of Cercopithecidae with an accentuated sulcus arcuatus only, or with a sulcus principalis. The gyral pattern of macaque monkey is close to that of *Cebus* monkeys and other Cercopithecidae with an accentuated sulcus arcuatus only, or with a sulcus principalis.

The Cebidae family of monkeys are mostly diurnal, but one genus, the Aotus, is primarily active at night. The oculomotor cortex of Cebidae has not been studied as widely or over such a long period of time as that of other monkeys or apes. It will be presented here in more detail. Compared to the gyral pattern of Cercopithecidae, the gyral pattern of Cebidae is variable. Owl monkeys (*Aotus trivarigatus*) are lissencephalic anterior to the central sulcus (Figure 3), as they only seldom have an inferior arcuate dimple (Huerta et al., 1986, 1987), and the oculomotor cortex is rather frontal. In squirrel monkeys (*Saimiri sciureus*) gyral variations range from no sulcus at all (Figure 3), to a simple dimple (Emmers and Akert, 1963; Huerta et al., 1987), to a small arcuate sulcus (Akert, 1964). The FEF is close to the dimple with considerable variation between individuals (Huerta et al., 1987). A small *dysgranular* area close to a particularly large dimple has been reported (Akert, 1964). The gyral pattern (Figure 3) of *Cebus* (*apella* or unspecified) is close to that of Cercopithecidae with a more accentuated sulcus arcuatus only (Sanides, 1970) or with a sulcus principalis (Tian and Lynch, 1997). As in the macaque, FEF in Cebus monkeys has been subdivided into two parts, one for smooth movement (FEMsem) and the other for saccadic movements (FEFsacc) (Tian and Lynch, 1996, 1997). In the Cebus, the FEFsacc is located at the apex of the arcuate sulcus on its anterior wall, a position close to that of Old World monkeys (Figure 3).

#### Apes

Hominoidea are a branch of Old World tailless anthropoid primates native to Africa and Southeast Asia. In comparison to the Cercopithecidae, major changes have occurred in the cortex of apes, especially in the areas anterior to the central sulcus. There is no sulcus resembling the arcuate sulcus of Cercipithecidae in apes. Differential studies carried out on a large number of chimpanzee brains showed considerable individual variation, particularly in the inferior precentral region (Sherwood et al., 2003). These authors insisted on the variability of the Broca’s area homologue in great apes in area 44 and had some difficulty in accurately identifying the inferior part of the precentral sulcus. They concluded that the inferior part of the precentral sulcus is not a reliable criterion for delimiting area 44. The location of the oculomotor area in chimpanzees has mainly been mapped using electrical stimulation (Grünbaum and Sherrington, 1903; Hines, 1940; Dusser de Barenne et al., 1941; Bailey et al., 1950). Despite these differences in cerebral sulcal patterns in chimpanzees, the position of the oculomotor area in the gorilla (Sherwood et al., 2003, 2004) and the orangutan (Beevor and Horsley, 1890) is about the same as that of the chimpanzee (Figure 7). Sherwood et al. (2004) suggested that this uniformity might reflect a common Bauplan^1^ to great ape brain macrostructural organization.

The individual variability of cerebral sulcal patterns makes it difficult to examine this idea closely. For, chimpanzees, gorillas and orangutans, the sulcus containing the oculomotor FD is always distant from the motor cortex, is *agranular* (von Bonin and Bailey, 1947), and has been functionally defined using microstimulation as homologous to the FD of macaques.

#### Humans

**Figure 4:**
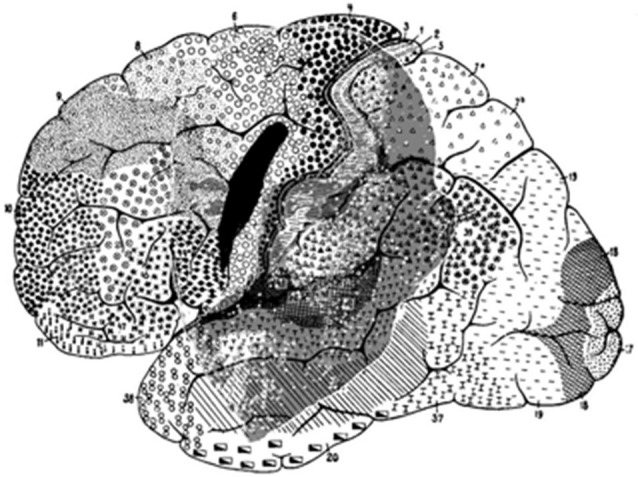
**Brodmann human brain areas defined and numbered based on the cytoarchitectural organization of neurons observed in the cerebral cortex using the Nissl stain**. Note that functional FEF does not denote regions that can be distinguished by the morphology of the cells contained within it.

There are major differences between apes and humans. However, even today, the location of the FEF in humans still raises intriguing problems. The human cerebral sulcal pattern is discernibly different from that of apes. The significant individual gyral variation, sometimes even between one hemisphere and the other, may partly explain the evident discrepancies in historical interpretations, which is particularly noticeable when comparing maps. Histologic studies have shown that the human FEF is not linked to any major sulcus (Pandya and Yeterian, 1996; Amiez et al., 2006) and that FEF is not located in Brodmann area 8 (Brodmann, 1909) (part of the granular cortex) but within the agranular cortex (Figure 4). Both PET and fMRI studies suggest that the activity of Brodmann area 8 is more associated with working memory, handling uncertainty, and analyzing coherent movements in the visual field, than eye movement *per se* (Cheng et al., 1995; Hyder et al., 1997; du Boisgueheneuc et al., 2006; Janata, 2009). Finally, some imaging studies have localized the human FEF in the precentral sulcus, abutting or within the primary motor strip.

**Figure 5:**
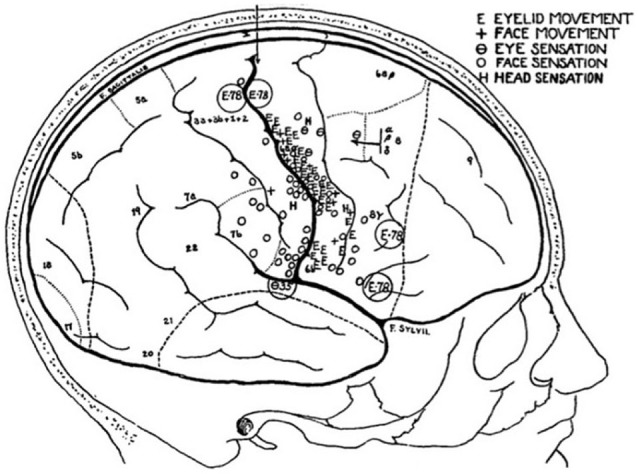
**Sketch of a patient’s brain, annotated throughout the operation, to show the areas that evoke sensations in, and movements of, the face (Penfield and Boldrey, 1937)**.

These imaging studies have tended to characterize the human FEF as mainly *precentral, premotor, and agranular* (Figure 5). According to these properties, the human FEF might have been associated with the premotor cortex in Cercopithecidae (area 6 of Brodmann, 1909), area FB of von Economo and Koskinas (1925), area 4 s of von Bonin (1949), between FA and FB for Bailey and von Bonin (1951), and in area 6 for Sarkissov et al. (1955). However, it is important to question the assumption that the measured neuronal activity is only related to moving the eyes (Kawashima et al., 1996). Images derived from control scans performed while subjects fixate are compared with images gathered during saccadic test scans. However, as Kawashima et al. (1996) mention, unless subjects are specifically instructed to inhibit blinking, it is common for blinking to occur when saccades are made. In contrast, subjects blink less frequently during steady fixation or at rest. It is therefore possible that imaging studies may have located the FEF too far caudally, toward a motor strip containing a region that mediates blinking responses. An imaging study designed to examine saccade generation failed to find the FEF near the expected precentral sulcus location. Instead insignificant precentral sulcus activation, with marked activation in the middle frontal gyrus was observed (Kawashima et al., 1996; Sugiura et al., 2004). An active region located in the middle frontal gyrus suggests anatomy homologous to monkey FEF. However, when Guipponi and colleagues tackled this question again recently, they showed that the identification of the neural correlates of spontaneous blinks in macaque monkeys does not map to the anterior bank of the arcuate sulcus (Guipponi et al., 2014). The measured fMRI activation has been identified as belonging to area 3b and not to motor primary cortex or to premotor area 6, calling into question the possible confounding factors revealed by previous studies. Evidence gathered using transcranial magnetic stimulation combined with structural MRI (Müri et al., 1991; Wessel and Kömpf, 1991) or imaging methods (Paus et al., 1996; Luna et al., 1998; Tehovnik et al., 2000) lends support to the location of the human FEF in the middle frontal gyrus.

#### Important Discrepancies

**Figure 6:**
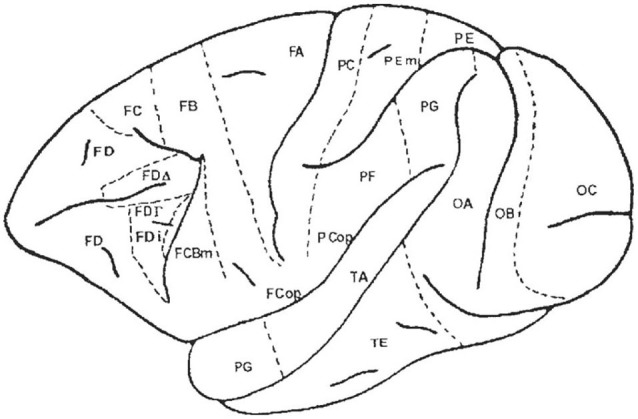
**Map of the macaque monkey cortex by von Bonin and Bailey (1947)**. Rostral view (left), lateral view (top). Note that von Bonin and Bailey designated most of the prefrontal cortex area FD, although they recognized two agranular areas, FF and FL.

In animal models, there is a major discrepancy between two major classical anatomical works. For Bailey and von Bonin (1951), macaque FDΓ is almost entirely buried in the inferior frontal sulcus, and is thus much smaller than von Economo’s area (Figure 6). None of the three areas—Brodmann’s area 8, von Economo’s area FdΓ, nor Bailey and von Bonin’s FdΓ—has a topographical relation with human FEF. Bailey and von Bonin (1951) noted that Brodmann’s work on the human brain was not extensive, and followed lengthy study of lemurs and monkeys. He published only a few figures of the human structures. Today, it is agreed that Brodmann’s human area 8 is not functionally homologous to simian area 8. Bailey et al. (1950) had already expressed doubt as to whether this area (in macaques) was homologous to FDΓ in the human brain. Foerster published on two occasions Foerster (1931, 1936) two maps drawn after direct stimulations in man. In his 1931 work, reproduced by Tehovnik et al. (2000) and redrawn by Brucher (1964), the FEF area is shown just in front of the precentral sulcus, close to the inferior frontal sulcus. This is approximately the position of area D that Dejerine (Dejerine and Roussy, 1906; Dejerine, 1914), claimed dealt with conjugate deviation of the head and eyes. The 2000 redrawing of Foerster’s (1936) map by Blanke and colleagues, places the eye field more medially, even crossing the superior frontal sulcus. Penfield and Rasmussen (1957) later showed that the sites that effectively stimulate eyelid movements or eye rotations were located more posteriorly just in front of the central sulcus or, more precisely, just in front of the motor cortex where movement of the arms, face, and mouth could be elicited (Figure 5). The only area of controversy was the evoked head movement that was sometimes located more anteriorly, close to, and around, the precentral sulcus. Penfield and Rasmussen’s (1957) localizations appear more posteriorly in comparison to recent maps (Chica et al., 2014). Blanke et al. (2000) also applied direct electrical stimulation to localize the FEF. They placed it in front of the precentral sulcus on both sides of the superior frontal sulcus, extending to the middle frontal gyrus. More recently, imaging methods have placed the human FEF in the precentral sulcus (Paus et al., 1996; Luna et al., 1998; Tehovnik et al., 2000).

Returning to histoanatomical data, the human FEF, as located by the recent histologic studies, is not located in Brodmann area 8 nor in von Economo FDΓ (Brodmann, 1909; von Economo, 1929; Huerta et al., 1987; Tehovnik et al., 2000; Amiez and Petrides, 2009). Surprisingly, the FEF of humans, in contrast to that of monkeys and apes, is no longer in the granular part of the cortex. Reviewing the data published on classical maps, FEF appears to be located within the agranular cortex—not in the giganto-pyramidal part of the motor cortex, but in the isocortex agranularis simplex of Bailey and von Bonin (1951). This region may be associated with the premotor cortex in Cercopithecidae: Brodmann’s (1909) area 6, von Economo and Koskinas’s (1925) area FB, von Bonin’s (1949) area 4 s, between FA and FB for Bailey and von Bonin (1951), and in area 6 for Sarkissov et al. (1955). This observation has also been made by Tehovnik et al. (2000). In all these species there are connections with the frontal cortex just anterior to, and within, the premotor cortex, just caudal to the FEF, though the connections to the supplementary motor area are controversial in macaques (Stanton et al., 1995) and differences in cortico-cortical connections have been reported between Cebidae and macaques (Huerta et al., 1987).

In addition, within the striatum, it is also known that in macaques the FEF sends axons to the dorsal part of the caudate nucleus (Künzle and Akert, 1977; Huerta et al., 1986; Stanton et al., 1988; Pouget et al., 2009) interspaced with the frontal granular islands, which are in the associative part of the striatum not the sensorimotor part. There is no indication that this would be the case in humans. In fact the thalamic territories where the largest differences between macaques and humans were observed, were those involved in oculomotor function. A recent review confirmed these observations by comparing diffusion tractography imaging of FEF-striatal motor pathways in humans and macaques (Neggers et al., 2015). The authors confirmed that in macaques FEF is connected with the head of the caudate and anterior putamen, and M1 is connected with more posterior sections of caudate and putamen, corroborating neuroanatomical tract tracing findings. In humans FEF and M1 are connected to largely overlapping portions of posterior putamen and only a small portion of the caudate. In that respect, some of the hypothetical differences between the cortico-subcortical connections with the FEF might explain some of the functional differences between the FEF in humans and macaques. This different position and histology of FEF in humans and macaques should discourage making of assumptions about its connections to other cortical and subcortical regions. Most of these connections in apes are unknown.

## Discussion

**Figure 7:**
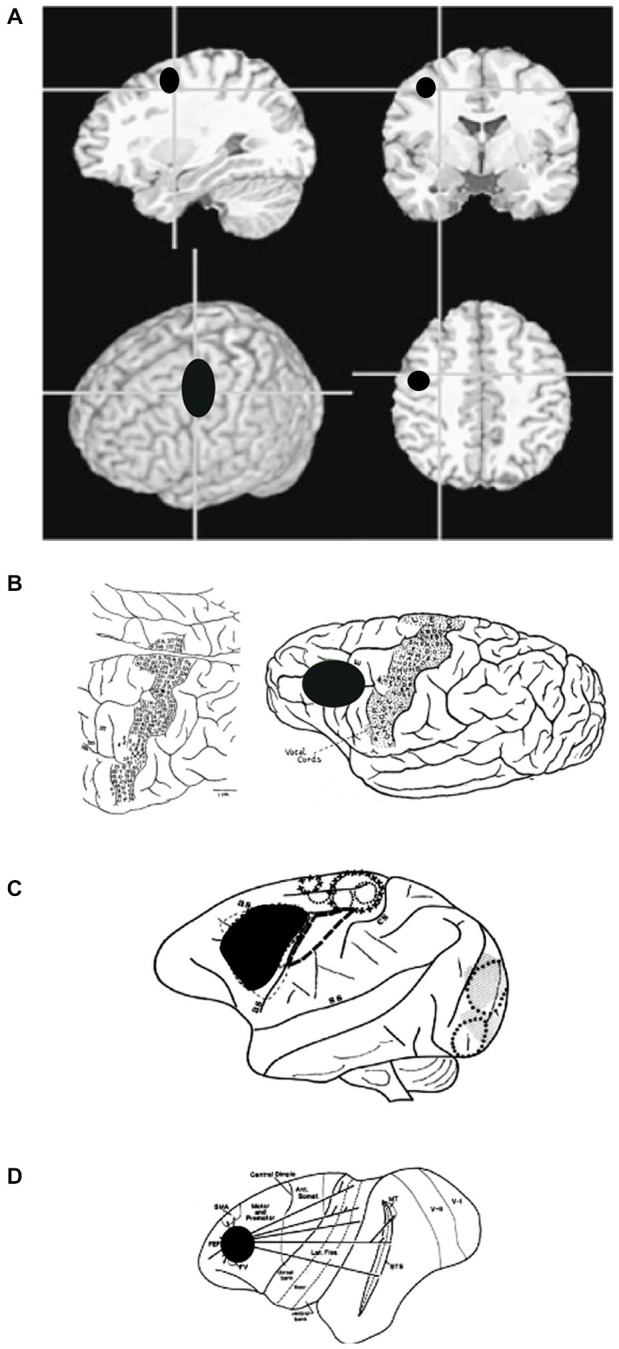
**(A)** Anatomical images showing the general location of FEF in humans. Note that human FEF appears to be more caudal compared to gorilla, macaque or owl monkey. **(B)** Sketch of a gorilla’s brain, annotated during surgery, to show areas that evoke motor responses in face, trunk, and eyes (Leyton and Sherrington, 1917). **(C)** Sketch of a macaque’s brain, annotated during surgery, to show areas that evoke eye movements. **(D)** Sketch of an owl monkey’s brain, annotated to show extrastriate areas that are connected to FEF.

One point is constant the FEF is always sulcal: to be more precise, it is located on one wall of the cerebral sulcus (Figure 7). This is obvious in Cercopithecidae that have a well delineated arcuate sulcus. In humans, it is located on the anterior bank of the superior precentral sulcus, close to the intersection between the precentral sulcus (vertical) and the superior frontal sulcus. This almost angular position is not sufficient to suggest that the superior precentral sulcus or the angle between this sulcus and the superior frontal sulcus is a remnant of the arcuate nucleus in Cercopithecidae (Blanke et al., 2000; Petrides and Pandya, 2002). Though the FEF of chimpanzees or gorillas is also sulcal, it has a different location; the sulcus does not evoke a transitory position and is does not lend itself to such an interpretation. Its sulcal position is likely due to the fact that its axons arrive early during the development and anchor in a still expanding cortex (e.g., Abeles, 1991).

The second point is the major change that has occurred between apes and humans. The FEF in apes is composed of a moderately thick and granular layer IV both rostrally and caudally. This granular layer IV becomes almost invisible at the fundus and into the posterior bank, whereas in humans, FEF is pre-central, pre-motor, and agranular. This makes it difficult to conclude that there is topological equivalence between the simian and human FEF position in the agranular—dysgranular—eugranular sequence. Leaving aside the location of the FEF in human, apes and macaques, these discrepancies between cytoarchitectonic classifications, together with the functional delimitations established with the use of fMRI, are raising fundamental questions about the importance of the topological position of cortical areas within the Sanides cytoarchitectonic gradients, but also on the core measures extracted from the fMRI technique by itself.

In 2000 Tehovnik and colleagues concluded that “the anatomy of FEFs is an enigma.” In 2015, we find ourselves sharing this view. Major studies in human and animals still need to be performed to reach any other position. Four questions still need to be answered (Tinbergen, 1951): firstly, how did the FEF evolve (phylogeny)? How does the FEF promote fitness (selection)? How does the FEF develop (ontogeny)? And finally, how does the FEF system work (mechanism)? Some elements of the first two questions have been addressed in comparative neuro-anatomy, where it has been found that the organization of the basal ganglia among birds, mammals and other vertebrates is similar. In contrast, the organization of the pallial domains of these groups is more varied (e.g., Jarvis et al., 2005). Some common pathways are also preserved in the sensorimotor domain. All vertebrates have a circuit dedicated to the processing of spatial sensory information and orienting responses, which is commonly centered on the optic tectum. Reptiles and birds have evolved a highly laminated optic tectum that is much more developed than the top-down control of the optic tectum in mammals. In particular the acropallial gaze fields are a major center in the bird gaze control circuitry, exerting top-down gain control of the brain stem spatial map via a parallel projection to the deep layers of the optic tectum and to the saccade-generating premotor neurons in the brain stem. In primates the projection to the superior colliculus of the FEF also exerts an indirect control on brain stem activity. Inter- and intra- species variability within these top-down oculomotor pathways have also been reported (Huerta et al., 1986; Tehovnik et al., 2000; Amiez et al., 2006). This review has mainly focussed on the displacement of the locus of FEF in primates, which lies on the arcuate sulcus in monkeys, but abuts the primary motor strip region in humans.

How does this displacement of FEF promote selection among primates? For a given species, how does the FEF develop ontogenetically? We believe that a synthesis of the responses to these questions holds the key to understanding the function of the FEF.

## Conflict of Interest Statement

The authors declare that the research was conducted in the absence of any commercial or financial relationships that could be construed as a potential conflict of interest.

## Footnotes

^1^Bauplan is a German word meaning an architectural plan. For FEF and primates, that meant a basic body plan for FEF structures and functions might have been modified by differences in sizes, proportions and by fusions and losses of other brain areas.
